# Flash Electroretinography and Pattern Visual Evoked Potential Changes in Ocular Hypertension Patients

**DOI:** 10.1155/2013/908017

**Published:** 2013-05-07

**Authors:** Ercüment Çavdar, Abdullah Ozkaya, Burcu Alper

**Affiliations:** ^1^Kemalpaşa State Hospital, Clinic of Ophthalmology, 35730 Izmir, Turkey; ^2^Beyoğlu Eye Training and Research Hospital, 34421 Istanbul, Turkey; ^3^Salihli State Hospital, Clinic of Ophthalmology, 45310 Manisa, Turkey

## Abstract

*Purpose*. To evaluate the changes of flash electroretinography (fERG) and pattern visual evoked potentials (pVEP) in ocular hypertension (OHT) patients. *Methods*. Twenty-five OHT patients and 30 healthy volunteers were enrolled for this cross-sectional study. Opthalmologic examinations, visual field tests, pVEP and fERG were performed. The main outcome measures were the differences between pVEP and fERG parameters. *Results*. The mean age of OHT patients and volunteers were 57 ± 12.25 years (range 30–65 years), and 53.25 ± 12.0 years (range 30–65 years), respectively. The mean amplitude of the pVEP was statistically lower in the OHT group (*P* < 0.05). Latency of the two groups was different; however, the difference was not statistically significant (*P* > 0.05). In fERG of OHT group, there was a significant decrease in the amplitude of the oscillatory potentials (Ops), and a significant delay in latency of rod and cone waves (all *P* < 0.05). There was no significant change in the flicker fERG waves between the two groups (*P* > 0.05). *Conclusions*. Although we found a decrease in Ops amplitude and a prolonged latency in flicker fERG, only the decrease in Ops amplitude was statistically significant between the two groups. The amplitude of Ops wave and amplitude of pVEP may reflect early glaucomatous damage in OHT patients.

## 1. Introduction

Glaucoma is an optic neuropathy which generally develops with elevated intraocular pressure (IOP) and leads to the enlargement of the cup disc ratio (C/D) in the optic nerve head (ONH) and visual field loss (VFL) [1, 2]. IOP measurement, assessment of ONH and visual field (VF), and examination of the anterior chamber angle are required in order to diagnose and classify glaucoma. Diagnostic criteria of glaucoma are highly variable. Glaucomatous VFL is one of the most important symptoms of glaucoma and frequently shows correlation with anatomic damage within the ONH. However, VF is affected when the axonal damage reaches 15%–50%, and this is the biggest disadvantage of VF testing [3].

Clinical electrophysiological tests allow us to assess the visual pathway as a whole system. Electrophysiological tests provide significant data while revealing and monitoring the pathologies in the visual pathway objectively from the retina pigment epithelium (RPE) to the occipital cortex [4].

A flash electroretinography (fERG) record is generated by the rapid changes of the retinal standing potential following retinal stimulation with a uniform light flash. It is thought that the formation of the waves during the record results from the outer retinal layers [4, 5]. The functional conditions of the retinal layers are defined with this record [6].

Histological studies reported that the increased reuptake of glutamine and synthesis of glial fibrillary acidic protein in Müller cells are associated whit ERG waves changes in glaucoma [7–10].

Pattern visual evoked potential (pVEP) is an electrical signal generated by the occipital visual cortex in response to the stimulation of the retina by pattern stimuli. These responses may provide information about the whole visual pathway from ganglion cells to the occipital cortex. The main purpose of pVEP is to obtain information regarding high afferent visual centers [11, 12].

Ocular hypertension (OHT) is defined as IOP greater than 21mmhg, no visual field defect, and no damage in ganglion cells [13]. However, conventional visual field tests remain normal until ganglion cell damage reaches 15%–50% and this results with confusion in the diagnosis and management of OHT. Therefore, alternative methods are needed for the diagnosis of OHT.

In this study, we aimed to evaluate the possible pVEP and fERG changes in patients with OHT in order to identify early ganglion cell damage.

## 2. Methods

Fifty-five patients were enrolled for this cross-sectional study between January 2008 and February 2009. The study and control group patients were recruited from the glaucoma department and the outpatient clinic. Approval for the study was obtained from the local ethics committee and performed in accordance with the tenets of Declaration of Helsinki.

The study group (SG) of 25 patients consisted of the patients who were under followup with the diagnosis of OHT in glaucoma department, and the control group (CG) of 30 patients constituted of the patients who were admitted to our outpatient clinic for refractive errors. 

The patients in the SG ranged in age from 30 to 65 years and had no glaucomatous VF defects as per the Hodapp-Parrish-Anderson (HPA) (source for HPA) grading system. Intraocular pressure measurements were made by Goldman applanation tonometry and were corrected according to the central corneal thickness. The inclusion criteria for SG were as follows: patients who had IOP of 22 mmHg or over for at least two measurements, which were made at different times, no changes in ONH (diffuse or local neuroretinal rim thinning, flame shape hemorrhage, enlargement of C/D, or nerve fiber layer defects), open anterior chamber angles, normal color vision, no systemic or ocular disorder other than glaucoma to effect VF, no refraction defect over 3.0 diopter as to spherical equivalent in refraction examination, and had best corrected visual acuity (BCVA) of 20/25 or better. None of them had eye surgery previously, pupil anomaly, or pupils bigger than 7 mm when dilated.

The participants in the CG ranged between 30 and 60 years of age and had no glaucomatous VF defects as per HPA grading system nor any ONH changes (diffuse or local neuroretinal rim thinning or flame shape hemorrhages, enlargement of C/D, and nerve fiber layer defects). Control group patients showed no systemic disorder, had no ocular problems rather than refractive errors, had no previous surgery, and had IOP of 21 mmHg or below. Finally, control patients had no pupil anomaly and pupils bigger than 7 mm when dilated; they had no refraction defects over 3.0 diopters as to spherical equivalent in refraction examination and had BCVA of 20/25 or better.

Clinical protocols and specifications outlined by the International Society for Clinical Electrophysiology of Vision (ISCEV) were used in pVEP and fERG records. Normal values of fERG were determined in median within the framework of ISCEV standards. Dynamic glaucoma 2 program of Interzeag Octopus Perimeter 101 device was used in VF assessment. The test's reliability criteria determined that fixation losses, false positive responses, and false negative responses should be below 15%. 

## 3. Electrophysiological Record

All electrophysiological tests were applied between 15 and 17 o'clock to prevent the possible diurnal effects upon the tests. A comprehensive ophthalmologic examination was performed for each patient. Electrophysiological test records were made under standard conditions by the same device system and the same physician (EC). The measurements were obtained by Tomey Primus 2.5 electrophysiology unit (TOMEY GmbH Am Weichselgarten 19a, 91058 Erlangen, Germany).

The measurements were performed in a silent, comfortable, and dark room. Alcohol was applied to the skin for eliminating the skin oils. Electrode cream was used to place the skin electrodes. Initially, a pVEP test was performed and test results were obtained monocularly. pVEP was recorded with full-field black and white reversing checkerboard which was displayed on a television screen. All electrodes were set up to an impedance of less than 5 kilo ohm (k*ω*). At least two recordings of responses to 64 stimuli were recorded. Monocular viewing was used. Checks subtended 30′ of visual angle, viewed at 0.5 m, and were 20° sized. Low and high frequency filters were, respectively, 1 and 100 Hz, and analysis time was 300 ms. The mean luminance of the stimulus was 46 candle/m² (cd/m²), the active electrode (o2) was placed 2 cm above the protuberantia, the reference electrode (f2) was attached to the left wrist, and the neutral electrode (fpz) was applied to the forehand. While the patient was looking at the fixation point of the checkered screen, the electrical potentials on the occipital cortex were recorded. When lid or peripheral artifacts were formed, the records were repeated. The patient's fixation was closely monitored. N75, P100, and P100-N135 amplitudes were assessed as well as the latencies of N75 forming after approximately 75 milliseconds (ms), P100 forming after approximately 100 ms, and N135 forming after approximately 135 ms.

After obtaining the pVEP records, 1% cyclopentolate and 1% tropicamide drops were applied for pupil dilatation. Following dark adaptation for 30 minutes, the fERG record was initiated. For fERG records, a neutral electrode (which is a ground electrode) was placed on the frontal region, reference electrodes were placed on the temporal region, and active corneal electrodes were placed on the conjunctival sac under a 15 watt red light source, utilizing ganzfeld stimulation with a maximum flash intensity of 2 cd/m². Electrical impedance was smaller than 5 k*ω* for all electrodes. Band-pass filter was between 0.3 Hz and 300 Hz with an amplification of 5 k while artifactual signals (blinks) were automatically removed.

Data was recorded after flash stimulation. The measurements were applied to both eyes automatically by means of the device's software. Rod responses, cone responses, maximal rod-cone responses, and oscillatory potential (OP) responses were received after 30 minutes of dark adaptation. A single flash cone response and 30 Hz flicker response were received after a 10-minute light adaptation. 

### 3.1. Statistical Analysis

For data analysis, SPSS 10.0 package (SPSS Inc., Chicago, IL, USA) was used and normality tests of the data were performed. Regarding the test results, “student t test” was used to compare the parameters between the two groups. The difference between the groups was calculated by Chi-square test. The confidence level was assessed at *P* < 0.05. 

## 4. Results

The mean age of the 25 SG patients was 52 ± 7.5 years (ranging between 30 and 65). The mean age of 30 CG patients was 53.2 ± 7.5 years (ranging between 30 and 65). The patients in the SG consisted of 12 males (48%) and 13 females (52%). The patients in the CG consisted of 15 males (50%) and 15 females (50%). There was not a statistically significant difference between the SG and CG in terms of age and gender (*P* > 0.05 and *P* > 0.05) (Table 1).

The mean IOP of the SG group was 23.05 ± 1.45 mmHg (between 22 mmHg and 25 mmHg) and the mean IOP of the CG was 13.7 mmHg ± 1.45 mmHg (between 12 mmHg and 17 mmHg). The difference between the two groups was statistically significant (*P* < 0.001). The mean vertical C/D in SG group was 0.36 ± 0.08 (ranging between 0.2 and 0.4), and the mean vertical C/D in the control group was 0.32 ± 0.06 (ranging between 0.2 and 0.4). The difference between the two groups was not statistically significant (*P* = 0.135) (Table 2). 

There was no statistically significant difference between two groups regarding the mean latency values of pVEP waves. However, the mean amplitude of the SG patients was significantly lower than the mean amplitude of the CG patients (*P* < 0.05) (Table 3).

Table 4 shows general fERG results. The SG had a significant delay in rod and cone latency responses and a decrease in OP amplitude (OPs1) (*P* < 0.05). There was no difference between flicker ERG responses. 

No statistically significant difference was found between amplitude and latency values of the study and control groups for fERG and pVEP by age groups (*P* > 0.05) (Table 5). 

## 5. Discussion

In previous electrophysiological studies, it was suggested that the exposures to intraretinal synapses in glaucoma would delay the cortical responses by affecting the postretinal synapses [14–16]. Recent studies also suggested that the dorsal lateral geniculate nucleus is affected both functionally and structurally in persons with OHT [17, 18]. The amplitude and latency of pVEP might be affected in OHT cases and decrease in the amplitude seems to be more significant [19]. In this study, when compared with the CG, there was a statistically significant decrease in the amplitude of pVEP in the SG (*P* < 0.05). 

In a study by Lan et al., the correlation between the pVEP and VF was evaluated, and they reported that there was no a significant correlation between them [20]. In another study by Nykanen and Raitta, the correlation between fVEP and the parameters of the VF was studied [21]. They stated that there was a correlation between P120 amplitude and average deviation; however, there was no correlation between the latencies and the parameters of VF. 

It is known that primary damage of glaucoma targets the ganglion cells [22]. Some studies have stated that a and b waves forming in fERG records are produced by external retinal layers and no change is seen at these waves in the cases of glaucoma. This information has led to the opinion that this test cannot be used in glaucoma diagnosis [22, 23]. However, in some recent studies, it was shown that there was amplitude and latency changes in scotopic and photopic ERG [24, 25]. OPs amplitudes decrease both in scotopic and photopic ERG and flicker response anomalies and decrease at photopic negative response (PhNR) amplitudes in glaucoma patients [25–30]. Korth et al. stated that they had found abnormalities at fERG parameters of patients with congenital glaucoma and that changes became evident in advanced glaucoma cases. Scotopic ERG, OPs, and PhNR originate from inner retinal layers like pERG; therefore, they are expected to be affected in glaucoma patients [31]. Machida et al. reported that S-cone PhNR is more sensitive to glaucoma than L- and M-cone PhNR [32]. 

Higher IOP levels may influence the electrophysiological tests more significantly than lower IOP levels [33]. Machida et al. suggested that acute IOP elevation results in retinal thinning and may cause ERG abnormalities due to decrease of choroid perfusion pressure, therefore change the electrical activity of the retina [34]. Buchi and Wachtmeister reported that they observed significant changes both in the wave amplitudes (decrease) and latencies (delay) in OHT cases [33, 35]. In our study, we did not find significant electroretinographic changes for both amplitudes and latencies of OHT cases, the same for the decrease of the OP wave amplitude. The reason of the difference between these results may be the recording conditions. It may also be due to the fact that within these studies, they assess eyes with high IOP and advance glaucomatous damage rather than using an assessment of OHT patients.

It is thought that OPs waves arise from inner retinal layers, and inner retinal layers are prone to high IOP levels [36]. Therefore, OPs waves are expected to be affected in glaucoma and OHT patients. In this study, OPs wave amplitudes in fERG were found to be decreased in OHT patients and this phenomenon supports the previous studies [37]. In addition, electrophysiological tests may reveal cell damage and ganglion cell dysfunction earlier than psychophysical tests [33, 37].

In conclusion, a significant decrease of amplitude in pVEP was detected in OHT patients in our study. Despite identification of decrease in OPs 1 wave amplitude in fERG with delay in wave of latencies and extension in wave of latency in flicker ERG, we have found that only the decrease in OPs 1 wave amplitude is remarkable when these parameters are assessed in OHT patients. When we compare the decrease of OPs 1 wave amplitude with amplitude decrease in pVEP, we have concluded that pVEP can give more valuable results in OHT cases. We think that decrease of pVEP wave amplitude in OHT patients will assist in establishing diagnosis. The weak sides of this study were as follows. The study was a cross-sectional study, the patients were not followed up, the changes were not evaluated by time, pERG or mfERG might be more accurate to assess the ERG changes in this group of patients, and the examinations and tests might have been combined with optical coherent tomography to identify retinal nerve fiber layer loss especially in the OHT group; however, only fundus examination was performed for optic disc evaluation.

Oscillatory potentials may be helpful for the diagnosis of glaucoma and OHT. Today, the methods used for OHT diagnose might remain incapable of establishing diagnosis. It has not set forth how much information should be provided by electrophysiological test parameters for OHT diagnosis yet. This issue will be elucidated with the studies to be carried out on cases suspected to have OHT. It is believed that the studies to be performed on this subject by electrophysiological tests, which are considered important in this study, will provide positive results.

## Figures and Tables

**Table 1:** Distribution of the groups by gender.

Patient group	Gender	Number	%
Study group	Male	12	48
	Female	13	52
	Total	**25**	**100**

Control group	Male	15	50
	Female	15	50
	Total	**30**	** 100**

**Table 2:** Comparison of intraocular pressure and vertical C/D.

	Study group	Control group	*P*
Intraocular pressure (mmHg)	23.05 ± 1.45	13,70 ± 1.45	<0,001
C/D	0.36 ± 0.08	0,32 ± 0.06	0,135

C/D: cup/disc ratio, *P*: *P* value.

**Table 3:** Pattern VEP findings.

	Patient group	Average	*n*	*t*	*P*
pVEP amplitude (*µ*v)	Study	10.24 ± 3.69	25	3.95	<0.001
	Control	14.02 ± 3.45	30		

pVEP latency (ms)	Study	107.65 ± 10.16	25	1.565	0.109
	Control	104.10 ± 6.12	30		

pVEP: pattern visual evoked potentials, ms: millisecond, *μ*V: millivolt, *n*: number of patients, *t*: time, *P*: *P* value.

**Table 4:** fERG findings.

Parameter	Study group	Control group	*t*	*P*
Maximal rod-cone response				
Latency (ms)				
A	18.5 ± 1.0	17.0 ± 0.8	0.430	0.65
B	42.0 ± 0.8	39.0 ± 0.4	0.395	0.72
Amplitude (*μ*V)				
A	122 ± 4.0	125 ± 3.5	0.705	0.52
B	190 ± 5.2	193 ± 5.8	0.500	0.65
Rod response				
Latency (ms)				
A	22.4 ± 6.0	19.7 ± 5.3	0.504	0.49
B	75.0 ± 5.6	71.0 ± 4.2	0.420	0.62
Amplitude (*μ*V)				
A	4.7 ± 3.1	4.8 ± 1.6	1.245	0.38
B	85.0 ± 2.5	83.5 ± 2.0	1.300	0.26
Cone response				
Latency (ms)				
A	18.8 ± 0.9	15.3 ± 1.5	0.705	0.54
B	33.0 ± 0.2	31.0 ± 0.1	0.525	0.45
Amplitude (*μ*V)				
A	19.0 ± 4.9	19.0 ± 3.4	0.497	0.60
B	70.5 ± 0.4	69.0 ± 0.2	0.350	0.28
Flicker response (30 Hz)				
Latency (ms)	29.1 ± 3.0	25.2 ± 2.1	3.330	0.10
Amplitude (*μ*V)	61.2 ± 19.1	60.5 ± 11.0	0.028	0.99
Oscillatory potentials (OPs)				
Latency (ms)				
P1 (OPs 1)	17.0 ± 0.9	15.0 ± 1.2	0.595	0.57
P2 (OPs 2)	24.1 ± 0.1	22.2 ± 0.2	0.514	0.45
P3 (OPs 3)	33.5 ± 0.2	33.0 ± 0.1	0.485	0.15
P4 (OPs 4)	44.2 ± 0.8	40.2 ± 0.6	0.750	0.75
Amplitude (*μ*V)				
P1 (OPs 1)	12.9 ± 5.8	23.3 ± 6.1	2.225	0.02
P2 (OPs 2)	38.2 ± 1.5	40.3 ± 3.2	0.250	0.35
P3 (OPs 3)	3.9 ± 0.2	4.1 ± 0.1	0.310	0.42
P4 (OPs 4)	4.8 ± 0.5	5.1 ± 0.4	0.540	0.24

fERG: flash electroretinogram, ms: millisecond, *μ*V: millivolt, OPs: oscillatory potentials, P1–4: oscillatory potential measurements, *t*: time, *P*: *P* value.

**Table 5:** Statistical assessment of fERG (maximal combined response) and pVEP measurements of the groups by age.

		*n*	Wave type	Average	SD
Study group	Maximal combined response amplitude (*μ*V)	25	A	126.1	14
			B	190.5	19.5
	Maximal combined response latency (ms)	25	A	21.3	8.2
			B	45.0	8.6
	pVEP amplitude (*μ*V)	25		11.9	5.0
	pVEP latency (ms)	25		111.7	4.1

Control group	Maximal combined response amplitude (*μ*V)	30	A	128.2	46.3
			B	193.2	18.7
	Maximal combined response latency (ms)	30	A	22.9	14.9
			B	43.6	11.4
	pVEP amplitude (*μ*V)	30		11.3	1.4
	pVEP latency (ms)	30		108.0	6.7

pVEP: pattern visual evoked potentials, ms: millisecond, *μ*V: millivolt, *n*: number of patients, *t*: *t* value, SD: standard deviation, fERG: flash electroretinography.
